# High-definition optical coherence tomography intrinsic skin ageing assessment in women: a pilot study

**DOI:** 10.1007/s00403-015-1575-x

**Published:** 2015-06-12

**Authors:** M. A. L. M. Boone, M. Suppa, A. Marneffe, M. Miyamoto, G. B. E. Jemec, V. Del Marmol

**Affiliations:** Department of Dermatology, Université Libre de Bruxelles, Hôpital Erasme, Meise, Belgium; Health Sciences Faculty, Department of Dermatology, Roskilde Hospital, University of Copenhagen, Roskilde, Denmark

**Keywords:** High-definition optical coherence tomography, Intrinsic skin ageing, Qualitative and quantitative assessment

## Abstract

Several non-invasive two-dimensional techniques with different lateral resolution and measurable depth range have proved to be useful in assessing and quantifying morphological changes in skin ageing. Among these, only in vivo microscopy techniques permit histometric measurements in vivo. Qualitative and quantitative assessment of chronological (intrinsic) age-related (IAR) morphological changes of epidermis, dermo-epidermal junction (DEJ), papillary dermis (PD), papillary-reticular dermis junction and reticular dermis (RD) have been performed by high-definition optical coherence tomography in real time 3-D. HD-OCT images were taken at the internal site of the right upper arm. Qualitative HD-OCT IAR descriptors were reported at skin surface, at epidermal layer, DEJ, PD and upper RD. Quantitative evaluation of age-related compaction and backscattered intensity or brightness of different skin layers was performed by using the plugin plot *z*-axis profile of ImageJ^®^ software permitting intensity assessment of HD-OCT (DICOM) images (3-D images). Analysis was in blind from all clinical information. Sixty, fair-skinned (Fitzpatrick types I–III) healthy females were analysed retrospectively in this study. The subjects belonged to three age groups: twenty in group I aged 20–39, twenty in group II aged 40–59 and twenty in group III aged 60–79. Only intrinsic ageing in women has been studied. Significant age-related qualitative and quantitative differences could be noticed. IAR changes in dermal matrix fibers morphology/organisation and in microvasculature were observed. The brightness and compaction of the different skin layers increased significantly with intrinsic skin ageing. The depth of visibility of fibers in RD increased significantly in the older age group. In conclusion, HD-OCT allows 3-D in vivo and real time qualitative and quantitative assessment of chronological (intrinsic) age-related morphological skin changes at high resolution from skin surface to a depth of the superficial reticular dermis.

## Introduction

Skin ageing has become an important health market [34]. Many of the treatments offered claim to modulate processes involved in skin ageing. Testing the efficacy of these therapies is important for consumers, dermatologists, cosmetic industry and regulatory authorities [36, 44].

For the assessment of molecular mechanisms involved in intrinsic skin ageing, invasive tests are clearly the gold standard [2, 32, 50]. However, the invasive nature of skin biopsy is not the most suitable method to investigate skin ageing in the general population. Skin ageing is a physiological process and, for obvious ethical reasons, efficacy testing of anti-ageing treatments should therefore be based on non-invasive methods [11].

Several non-invasive two-dimensional imaging techniques with different lateral resolution and measurable depth range have proved to be useful in assessing and quantifying morphological changes in skin ageing [1, 10]. Among these, only in vivo microscopy techniques such as reflectance confocal microscopy (RCM) [30, 40, 42, 51] and multiphoton laser scanning tomography (MPT) [21, 23, 24, 38] permit cellular resolution in vivo. In vivo techniques without cellular resolution dealing with skin ageing are high-frequency ultrasound (HF-US) [16–19, 41, 43, 49] and conventional optical coherence tomography (OCT) [13, 25, 31, 33, 35].

High-definition OCT (HD-OCT) is a recently introduced non-invasive technology based on the principle of low coherence interferometry [4–9, 15]. This method permits real time three-dimensional (3-D) imaging with cellular resolution up to 570 µm depth. This enables visualization of cells in their micro-architectural environment at up to the superficial reticular dermis. It was recently demonstrated that real time 3-D imaging provides accurate information on dermal matrix fibre organisation and microvasculature volume [9].

The aim of this study is the qualitative and quantitative 3-D HD-OCT assessment of intrinsic age-related (IAR) morphological skin changes [46] from skin surface up to the superficial reticular dermis.

## Methods

### Study sample

HD-OCT images of 60, fair-skinned, healthy females (skin types I–III) were retrieved from first author’s private practice for inclusion in this retrospective study. Inclusion criteria were (1) availability of good quality HD-OCT images of normal skin at inner site of upper arm and (2) absence of signs of actinic damage in this anatomic region. These images were taken during daily practice as control/reference HD-OCT images in comparison with HD-OCT images of affected skin. We conformed to the Helsinki Declaration with respect to human subjects in biomedical research. All international rules governing clinical investigation of human subjects were strictly followed. Approval from local ethical committee and informed consent from all participants were obtained. Moreover, this study affected neither the routine diagnosis nor treatment of the lesions presented by the included subjects.

### Image acquisition by HD-OCT

Non-invasive 3-D imaging by HD-OCT (Skintell^®^, AgfaHealthcare, Mortsel, Belgium) has been used to image the internal site of the right upper arm by holding the probe aligned with the axis of the humerus. Instruments and acquisition methods have been previously described [4–9].

### Evaluation of IAR morphological HD-OCT features

All images were evaluated by first author, in blind from any clinical information.

Following *Z*-levels were scanned: skin surface, dermo-epidermal junction (DEJ), upper papillary dermis (up-PD), lower papillary dermis (low-PD) and upper reticular dermis (up-RD). The junction between PD and RD is represented by the highest peak after the valley [33].

#### Qualitative evaluation

##### Standard colour setting (Table 1; Fig. 1)

**Table 1:** Absolute and relative frequencies of intrinsic skin ageing related morphologic parameters imaged by 3-D HD-OCT in standard colour setting

	Young-aged group (*N* = 20)	Middle-aged group (*N* = 20)	Old-aged group (*N* = 20)
Furrow pattern			
Small rhomboidal	19 (95.0 %) (*p* < *0.001*)*	1 (5.0 %)	0 (0.0 %)
Large rhomboidal	1 (5.0 %)	11 (55.0 %) (*p* < *0.001*)	1 (5.0 %)
Linear	0 (0.0 %)	7 (35.0 %) (*Not significant*)	5 (25.0 %)
Disarranged	0 (0.0 %)	1 (5.0 %)	14 (70.0 %) (*p* < *0.001*)
Flattening			
Cross-sectional			
Jagged	20 (100.0 %) (*p* < *0.01*)	15 (75.0 %)	0 (0.0 %)
Flat	0 (0.0 %)	5 (25.0 %)	20 (100.0 %) (*p* < *0.001*)
En face			
Papillary rings present	20 (100.0 %) (*p* < *0.01*)	15 (75.0 %)	0 (0.0 %)
Irregular rings	1 (5.0 %)	10 (50.0 %) (*p* < *0.01*)	0 (0.00 %)
Papillary rings absent	0 (0.0 %)	5 (25.0 %)	20 (100.0 %) (*p* < *0.001*)

* *p* values have been mentioned whenever appropriate; for details see “Results”

**Fig. 1:**
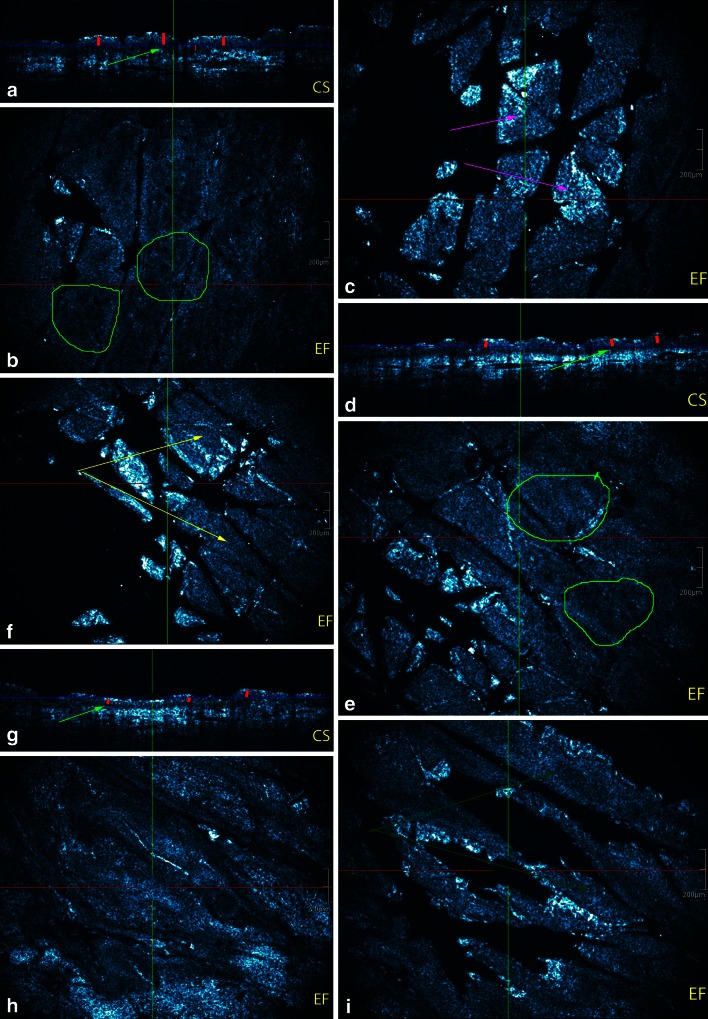
Intrinsic age-related morphological parameters imaged by HD-OCD in en face (EF) and cross-sectional (CS) mode. **a**–**c** Young women: a small rhomboidal furrow pattern is noticed (*magenta arrow*). ET (*red vertical lines*) is >60 µm. DEJ is jagged on cross-sectional image (*green arrow*) and papillary rings are regular and small (*green circle*). DEJ is thickest in young adult (*orange line*). **d**–**f** Middle aged women: less intersecting furrows are noticed resulting in larger rhomboidal furrow pattern (*yellow arrows*). ET (*red lines*) is lesser compared to young adult. More flattening of the DEJ is observed on cross-sectional image (*green arrow*). Papillary rings are larger and more irregular on en face imaging (*green circle*). DEJ is thinner compared to young adult. **g**–**i** Older aged women: a *disarranged furrow pattern* is observed with some linearization of the furrows (*dark green* *arrows*). ET is thinnest in this group (*red vertical lines*). The DEJ is flat and very thin (*green arrow*). This results in almost absence of papillary rings on en face image

1. Furrow patterns have been evaluated as previously described [51]: small rhomboidal, large rhomboidal, linear or disarranged at skin surface on en face images,
2. Flattening (effacement) of DEJ on cross-sectional images and altered ringed pattern of papillary rings on en face images [30]

##### Inverted colour setting: fibers assessment (Table 2; Figs. 2, 3, 4)

**Fig. 2:**
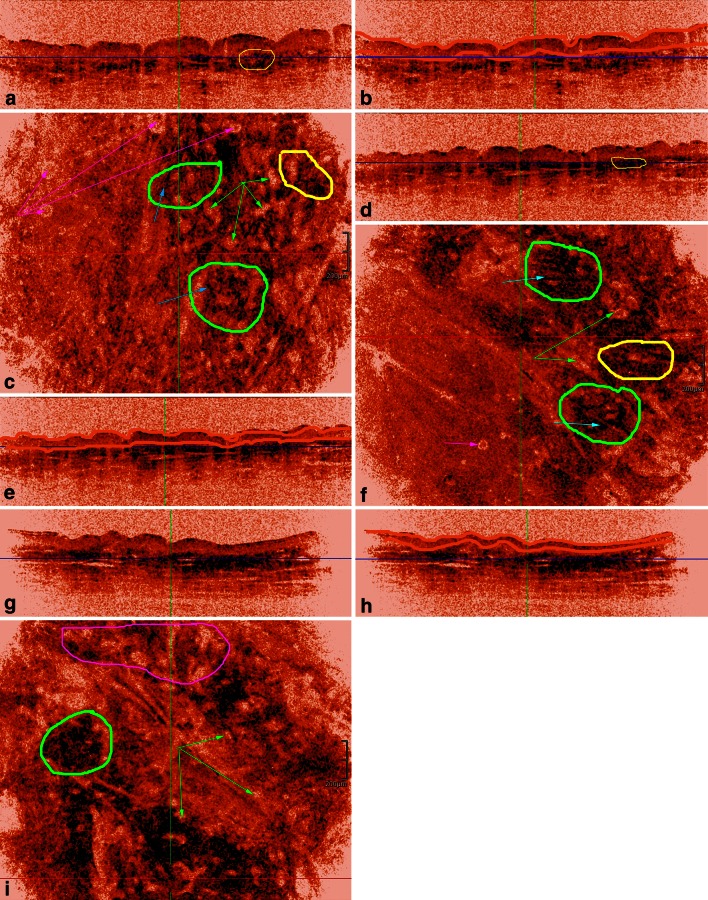
HD-OCT dermal ultrastructural and microvascular features of PD in women of three different age groups. Young women: **a** cross-sectional image with *dark blue horizontal line* indicating the *Z* value of the en face image. **b** Same cross-sectional image with epidermis edging (*orange lines*). Left side of the *blue line* is still corresponding to DEJ while right side of the *blue* *line* is already corresponding to the PD. **c** Corresponding en face image. Left side displays the DEJ and some artefacts (*magenta arrows*). Right side displays the superficial part of the PD. Thin, short and curled fibers form a randomly oriented feltwork (*green circles*). *Disperse* *black dots* align with these fibers (*light blue arrow*). *Clusters of black dots* (*yellow circles*) are noticed around the capillary loops (*green arrows*). The capillary bed of PD present as numerous homogenous distributed small round hyporeflective spaces (*green arrows*). Candle stick holder configuration of black dots is encircled in *yellow* on cross-sectional image. Middle aged women: **d** cross-sectional imaging with dark blue horizontal line indicating the *Z* value of the en face image. **e** Same cross-sectional image with epidermis edging (*orange lines*). Left side of the *blue line* is still corresponding to the DEJ. Right side of the *blue* *line* is already indicating the PD. The ET is thinner than in young adults. **f** Corresponding en face image. 2/3 of left side displays the DEJ with one artefact (*magenta arrow*). Right side displays the superficial part of the PD. Fibers are thicker, longer and straighter compared to young adults. Moreover, they become more aggregated and aligned in few directions (*green circles*). Disperse *black* *dots* condensed but still aligned with fibers (*light blue arrows*). Clusters of *black* *dots* increase in density and are progressively oriented in a more horizontal way (thin *yellow circle* on cross-sectional image). A reduction of the capillary area is observed (*green arrows*). Older aged women: **g** cross-sectional image with *dark* *blue line* indicating the *Z* value of the en face image. **h** Same cross-sectional image with epidermis edging (*orange lines*). At the left side of the image the *blue* *line* corresponds with lower PD while at the right side of the image *blue line* corresponds with middle part of PD. Epidermal thickness is thinner than in middle aged adults. **i** Fibers become more and more aggregated in a lace-like network (*green circle*). Clusters of *black dots* merge to form compact *dark blotches*. A further reduction of the density of the capillary bed (*green arrows*) is noticed resulting in an increase in lower capillary density. The top of the image corresponds with the upper part of the reticular dermis displaying a prominent sub-papillary plexus (*magenta circle*)

**Fig. 3:**
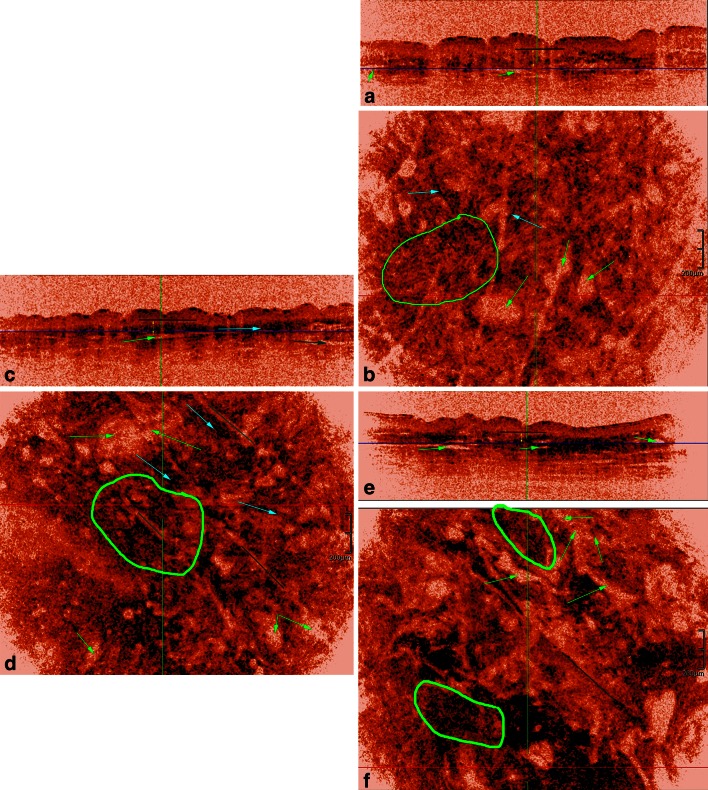
HD-OCT dermal ultrastructural and microvascular features at the junction of papillary and reticular dermis in women of three different age groups. Young women: **a** cross-sectional image with *dark blue horizontal line* indicating the *Z* value of the en face image. *Short black line* with *Z* value just under basal cell layer. The thickness of the PD is shown by a double *yellow arrow*. The sub-papillary plexus corresponds with larger, elongated and horizontal oriented hyporeflective spaces (*green arrows*). **b** Corresponding en face image displays a randomly oriented network of coarse, long, curved but discrete rope-like bundles of fibers (*green* *circle*). Disperse black dots align with these fibers (*light blue arrow*). Clusters of black dots are sparse. The sub-papillary plexus corresponds with larger, elongated and horizontal oriented hyporeflective spaces. (*green arrows*). Middle aged women: **c** cross-sectional image with *dark blue horizontal line* indicating the *Z* value of the en face image. *Short black line* with *Z* value just under the basal cell layer. The thickness of the PD is indicated by a *double yellow arrow*. The papillary thickness is smaller compared to young adults. **d** Corresponding en face image. Fibers are thicker, longer and straighter compared to young adults. They form more marked rope-like bundles of fibers (*green circle*). Moreover, they become more aggregated and aligned in few directions corresponding with furrow pattern (*dark green lines*). *Disperse* *black dots* condensed but still aligned with fibers (light blue arrows). *Clusters of black dots* increase in density and are progressively oriented in a more horizontal way (*light blue arrow*). The sub-papillary plexus corresponds with larger, elongated and horizontal oriented hyporeflective spaces. (*green arrows*). Older aged women: **e** cross-sectional image with *dark blue horizontal line* indicating the *Z* value of the en face image. *Short black line* with *Z* value just under the basal cell layer. The thickness of the PD is indicated by a *double yellow arrow*. The papillary thickness is smallest. **f** Corresponding en face image. Straight rope-like bundles of fibers (*green circle*) aggregate and align in one main direction corresponding with the furrow pattern (*dark green lines*). The Clusters of *black dots* merge to form compact dark blotches. The sub-papillary plexus becomes very prominent with strongly dilated horizontal oriented elongated branching hyporeflective spaces (*green* *arrows*)

Bright structures in the standard colour setting appear dark in the inverted colour setting permitting better assessment of fibers. In addition, collagen fibers cannot be distinguished from elastic fibers by HD-OCT as previously shown [9] (Figs. 2, 3).

**Table 2:** Absolute and relative frequencies of intrinsic skin ageing related morphological features of dermal matrix fibers and dermal microvasculature imaged by 3-D HD-OCT in inverted colour setting

	Young-aged group (*N* = 20)	Middle-aged group (*N* = 20)	Old-aged group (*N* = 20)
Dermal matrix fibers			
Morphology			
Up-PD: curled thin short fibers	19 (95.0 %) (*p* < *0.001*)*	8 (40.0 %)	0 (0.0 %)
Up-PD: curled thick fibers	1 (5.0 %)	12 (60.0 %)	20 (100.0 %) (*p* < *0.01*)
Low-PD: thick, intermediate wavy fibers	19 (95.0 %) (*p* < *0.001*)	6 (30.0 %)	0 (0.0 %)
Low-PD: thick straight fibers	1 (5.0 %)	14 (70.0 %)	20 (100.0 %) (*p* < 0.01)
Up-RD: coarse intermediate curved rope-like bundles of fibers	18 (90.0 %) (p < 0.001)	7 (35.0 %)	0 (0.0 %)
Up-RD: coarse intermediate/long straight rope-like bundles of fibers	2 (10.0 %)	13 (65.0 %)	20 (100.0 %) (*p* < *0.01*)
Organisation of fibers			
Up-PD: short fibers loosely interwoven	19 (95.0 %) (*p* < *0.001*)	6 (30.0 %)	0 (0.0 %)
Up-PD: fibers aggregating in lace-like network	1 (5.0 %)	14 (70.0 %)	20 (100.0 %) (*p* < *0.01*)
Low-PD – Up-RD: fibers in randomly “feltwork”	19 (95.0 %) (*p* < *0.001*)	5 (15.0 %)	0 (0.0 %)
Low-PD – Up-RD: intermediate fibers aligned in few directions	1 (5.0 %)	13 (75.0 %) (*p* < *0.01*)	6 (30.0 %)
Low-PD – Up-RD: long fibers aligned in one direction corresponding with furrow pattern	0 (0.0 %)	2 (10.0 %)	14 (70.0 %) (*p* < *0.01*)
Clusters of dots in papillary dermis			
Dispersed aligned with fibers or lining hyporeflective holes in vertical orientation “Candle stick holder”	19 (95.0 %) (*p* < *0.001*)	4 (20.0 %)	0 (0.0 %)
Increased density (horizontal orientation-condensed)	1 (5.0 %)	16 (80.0 %) (p < 0.001)	2 (10.0 %)
Compact blotches	0 (0.0 %)	0 (0.0 %)	18 (90.0 %) (*p* < *0.001*)
Blood vessels (hyporeflective spaces)			
Capillary density in papillary dermis			
High	19 (95.0 %) (*p* < *0.001*)	2 (10.0 %)	0 (0.0 %)
Intermediate	1 (5.0 %)	17 (85.0 %) (*p* < *0.001*)	2 (10.0 %)
Low	0 (0.0 %)	1 (5.0 %)	18 (90.0 %) (*p* < *0.001*)
Vessel morphology in papillary dermis			
Large ovoid	19 (95.0 %) (*p* < *0.001*)	3 (15.0 %)	0 (0.0 %)
Small ovoid	1 (5.0 %)	16 (80.0 %) (p < 0.001)	1 (5.0 %)
Small round “pinholes”	0 (0.0 %)	1 (5.0 %)	19 (95.0 %) (*p* < *0.001*)
Vessel morphology in reticular dermis			
Small elongated in horizontal plane	19 (95.0 %) (*p* < *0.001*)	2 (10.0 %)	0 (0.0 %)
Large elongated in horizontal plane	1 (5.0 %)	18 (90.0 %) (*p* < *0.001*)	2 (10.0 %)
Prominent and branched vessels with hyper-reflective cuff	0 (0.0 %)	0 (0.0 %)	18 (90.0 %) (*p* < *0.001*)

p values in italic are significant if *p* < 0.05

*Up-PD* upper papillary dermis, *Low-PD* lower papillary dermis, *Up-RD* upper reticular dermis

* *p* values have been added whenever appropriate; for details see “Results”

**Fig. 4:**
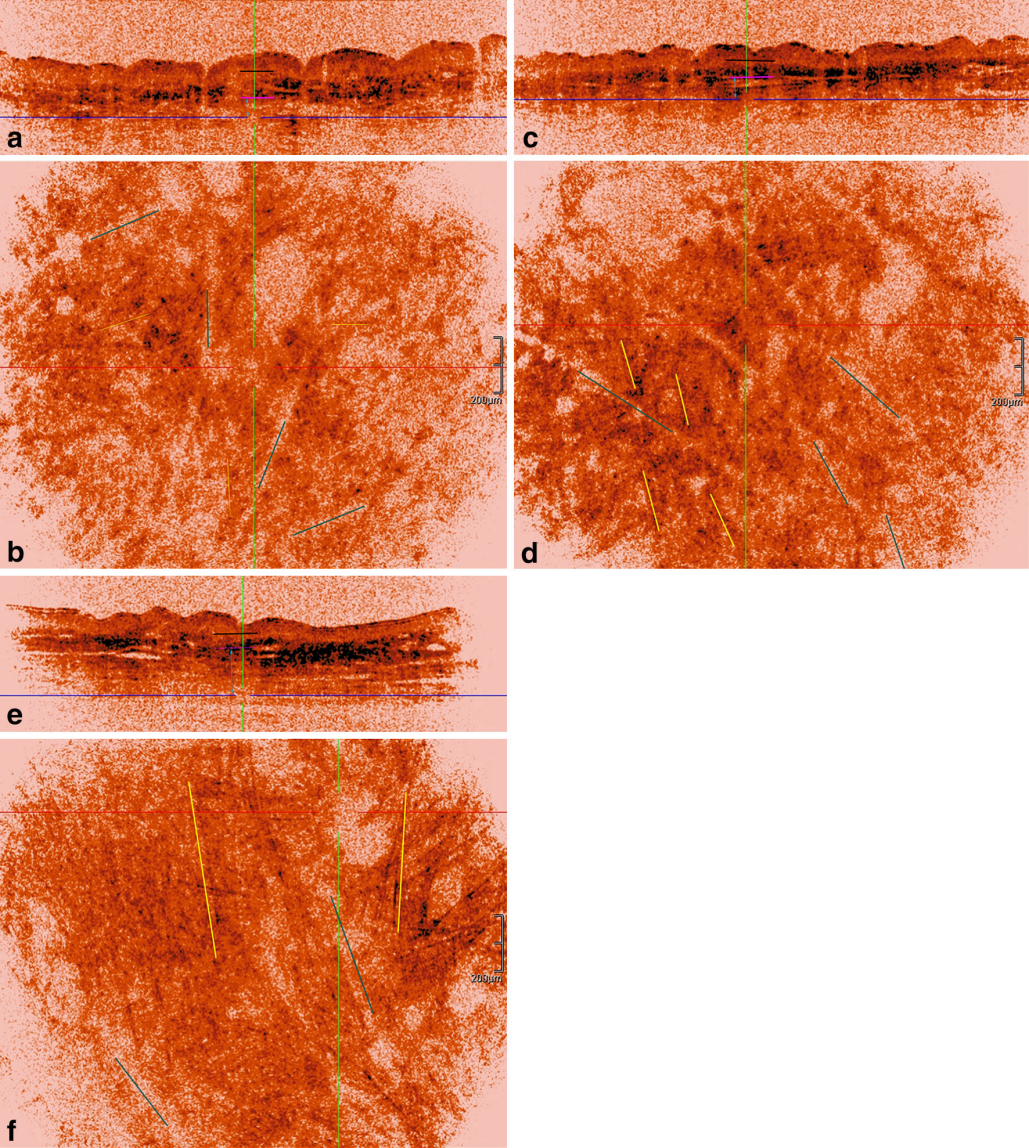
HD-OCT imaging of dermal fibers deeper in reticular dermis in women of three different age groups. Young women: **a** cross-sectional image with *dark blue horizontal line* indicating the *Z* value of the en face image. *Short black line* with *Z* value just under basal cell layer. *Short magenta line* with *Z* value corresponding with junction of papillary and reticular dermis. Fibers are still visible up to a depth of 80 µm under the junction of papillary and reticular dermis (*light blue double* *arrow*). **b** Corresponding en face image displays a randomly oriented network of discrete rope-like bundles of fibers (*yellow lines*). *Furrow lines* are displayed (*green lines*). Middle aged women: **c** cross-sectional image with *dark blue* *horizontal line* indicating the *Z* value of the en face image. *Short black line* with *Z* value just under basal cell layer. *Short magenta line* with *Z* value corresponding with junction of papillary and reticular dermis. Fibers are still visible up to a depth of 65 µm under the junction of papillary and reticular dermis (*light blue double arrow*). **d** Corresponding en face image. Fibers form marked rope-like bundles. They become more aggregated and aligned in few directions corresponding with furrow pattern (*dark green lines*). Older aged women: **e** cross-sectional image with *dark blue horizontal line* indicating the *Z* value of the en face image. *Short black line* with *Z* value just under basal cell layer. *Short* *magenta line* with *Z* value corresponding with junction of papillary and reticular dermis. Fibers are still visible up to a depth of 185 µm under the junction of papillary and reticular dermis (*light blue double arrow*). **f** Corresponding en face image. Straight rope-like bundles (*yellow lines*) align in almost one direction. This predominant direction corresponds with the linear furrow pattern (*green dark* *lines*). Very long fibers (>400 µm) are observed

1. *Thickness of the fibers* Thin (<12 µm), thick (>12 and <18 µm), coarse (>18 µm),
2. *Length of the fibers* Short (<80 µm), intermediate (between 80 and 300 µm) and long (>300 µm),
3. *Morphology of the fibers* Curled, wavy, curved or straight rope-like.
4. *Organisation of the fibers* Loosely interwoven, aggregating in lace-like network (PD) or in randomly “feltwork” (RD), aligned in few or in one direction(s).
5. *Clusters of dark dots* (a) Dispersed, aligned with fibers or lining hyporeflective holes in vertical orientation (“Candle stick holder”-distribution) (b) increased density (condensed) with horizontal orientation or (c) compact blotches.

##### Inverted colour setting: dermal microvasculature assessment at two levels: capillary bed in the PD and sub-papillary vascular plexus in RD

The inverted colour setting permitted better assessment of microvasculature. The cutaneous microvasculature presented as hyporeflective spaces (Figs. 3, 4).

1. *Capillary density in PD* High, intermediate or low
2. *Morphology of hyporeflective spaces in PD* Large ovoid, small ovoid or small round “pinholes”
3. *Morphology of hyporeflective spaces in upper RD* Small elongated in horizontal plane, large elongated in horizontal plane or prominent and branched vessels with hyperreflective cuff.

#### Quantitative evaluation

**Fig. 5:**
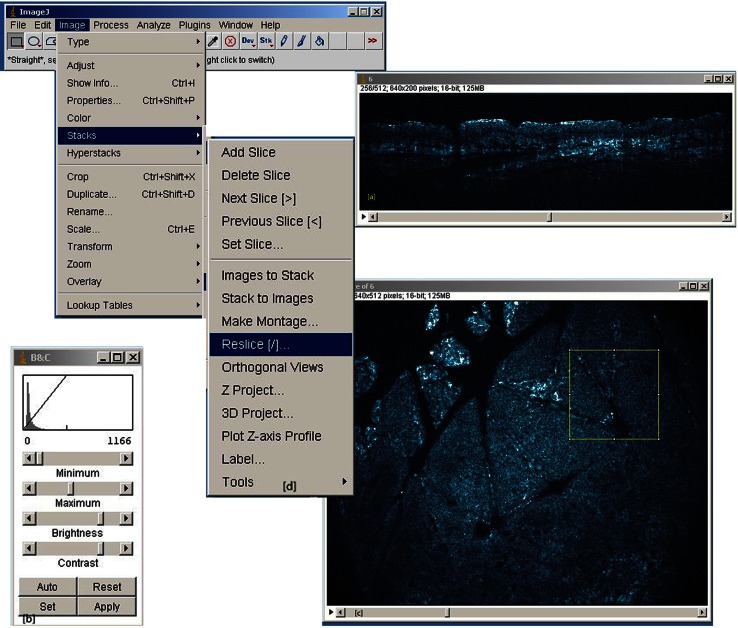
Plugin plot *z*-axis profile of ImageJ^®^ software analysis of 3-D HD-OCT DICOM images. Procedure is as follows, **a** 3-D Dicom image (128 MB) of interest is selected and file opened as cross-sectional view. **b** Correction for brightness and contrast (minimum “0” and maximum value is chosen between “1150 and 1200”). **c** Image > Stack > Reslice of image in order to open the corresponding en face image. Four square (450 × 450 µm) regions of interest (ROI) were chosen, one in each quadrant of the horizontal (en face) image. **d** Image > Stack > plot *z*-axis profile: Intensity (I) of brightness of single VOIs (Volume of interest: scanned volume equals 450 µm × 450 µm × 570 µm = 0.11 mm^3^) is assessed and values transferred to excel table and displayed in a graphic (Fig. 6)

Backscattered intensity assessment of 3-D HD-OCT DICOM (digital imaging and communication in medicine) images was achieved by using the plugin plot *z*-axis profile of ImageJ^®^ software. This is an open source image processing program designed for scientific multidimensional images. The procedure is explained in Fig. 5.

**Fig. 6:**
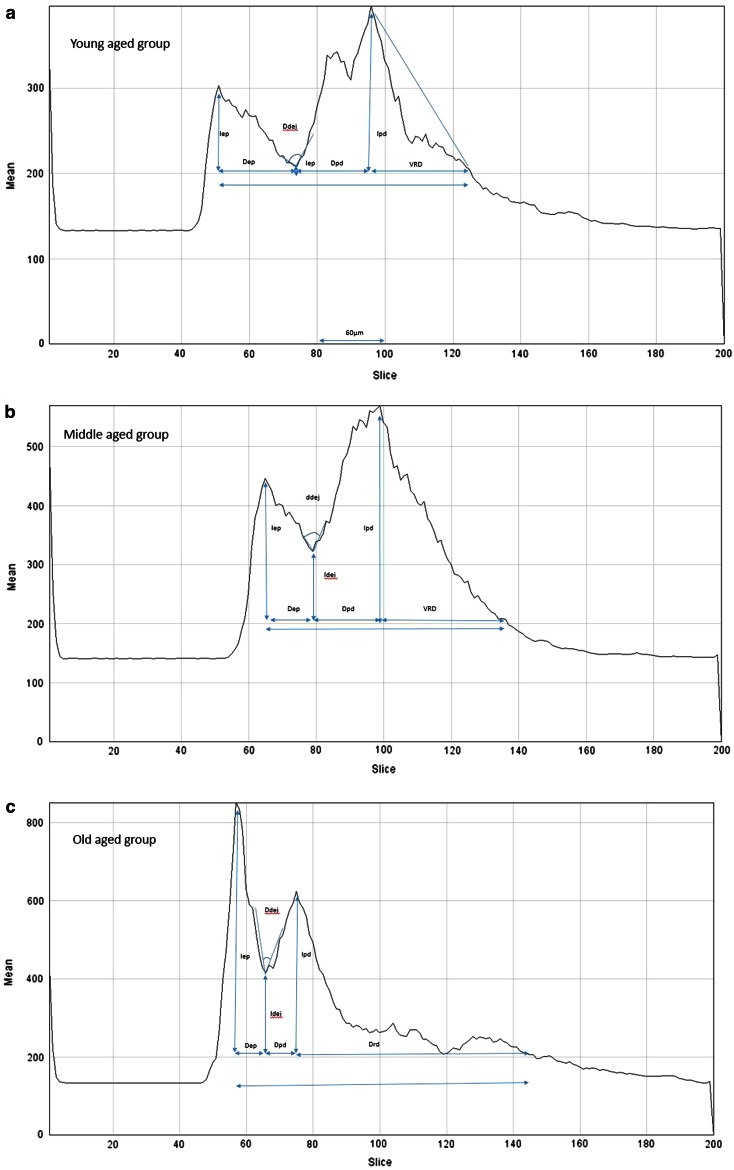
Evaluation of compaction (measured on the *x*-axis: from 0 to 200; 1 unit = 3 µm; axial position or depth) and brightness (backscattered intensity) (*y*-axis from 0 to 800 AU (arbitral units). *Compaction of epidermis:* (Δ_EP_ = distance from first peak to the valley’s middle point in µm) and *brightness of epidermis* (*I*_EP_ = difference between first peak and the 200 AU line: under this value only noise could be detected on the original DICOM image). *Compaction* *of DEJ* (Δ_DEJ_ = angle (in degrees) produced by the descendent line of the valley and the ascendant line of the valley) and *brightness of DEJ* (*I*_DEJ_ = difference between the bottom of the valley and the 200 AU line). *Compaction* *of PD* (Δ_PD_ = distance from middle of valley to second peak in µm) and *brightness of PD* (*I*_PD_ = difference between the highest peak after the valley and the 200 AU line). The highest peak after the valley represents the junction between PD and RD [33]). *Depth of visibility of fibers in RD* (*V*_RD_ in µm): under the 200 AU line only noise could be detected. **a** Compaction and brightness in young-aged skin (for discussion see “Results”). **b** Compaction and brightness in middle-aged skin (for discussion see “Results”). **c** Compaction and brightness in old aged skin (for discussion see “Results”)

The quantitative evaluation included compaction (measured on the *x*-axis: from 0 to 570 µm axial position or depth) and evaluation of backscattered intensity “brightness” (measured on the *y*-axis from 0 to 800 arbitral units (AU). (see Fig. 6 for details). In addition the depth of visibility of fibers in RD was measured. The junction between PD and RD is represented by the highest peak after the valley [33].

### Statistical analysis

One-way analysis of variance (ANOVA) was used to compare means of three samples using the F distribution. Moreover, Scheffé test was used for all pairwise comparisons. Calculations were made by using MedCalc^®^ statistical software version 14.12.0.

All dichotomous variables describing the presence/absence of particular HD-OCT features of skin ageing were included. Absolute and relative frequencies were calculated for different age groups. Chi-squared (*χ*^2^) test was employed to compare each age group versus the other age groups. The phi (*φ*) coefficient, employed to weight diagnostic power of each significant parameter, is a measure of association of two binary variables and is related to the Chi-squared (*χ*^2^) statistic by the formula: *φ*^*2*^ = *χ*^2^/*n,* where *n* equals the total number of observations.

## Results

### Subjects

Sixty, fair-skinned, healthy females were analysed retrospectively for this study. The patients belonged to three age groups: 20 in group I aged 20–39 (Young-Aged: YA), 20 in group II aged 40–59 (Middle-Aged: MA) and 20 in group III aged 60–79 (Older-Aged: OA).

### Qualitative evaluation of IAR morphological HD-OCT features

#### Standard colour setting (Table 1; Fig. 1)

##### The furrow pattern at skin surface on en face images differed according to age

Small rhomboidal furrow pattern was a sensitive (SS) and specific (SP) feature of YA-group (95 % and 97.5 %, respectively; *φ* = 0.93, *χ*^2^ = 51.34, *p* < 0.001). Large rhomboidal pattern and linear furrow patterns were co-dominant in the MA-group with moderate sensitivity but high specificity (large rhomboidal: 55 % SS and 97.5 % SP; *φ* = 0.62, *χ*^2^ = 22.97, *p* < 0.001 and linear furrow: 35 % SS and 87.5 % SP; *φ* = 0.27, *χ*^2^ = 0.47, NS). A disarranged furrow pattern was a sensitive and specific feature for the OA-group (70 and 97.5 %, respectively; *φ* = 0.73, *χ*^2^ = 32.4, *p* < 0.001). A large rhomboidal and linear pattern was also observed in the OA group in 1/20 and 5/20 cases, respectively.

##### Flattening of DEJ on cross-sectional images vs papillary ring on en face images

Flattening of DEJ was a highly sensitive and specific feature of the OA-group (99.5 % SS and 87.5 % SP; *φ* = 0.83, *χ*^2^ = 41.57, *p* < 0.001). A jagged subepidermal dark band was highly sensitive but moderately specific for the YA-group (99.5 % SS and 62.5 % SP; *φ* = 0.59, *χ*^2^ = 21.06, *p* < 0.01). Follicular structures could interrupt these images. However, on the corresponding en face images age-related alterations of papillary rings were observed: small regular rings in YA (99.5 % SS and 62.5 % SP; *φ* = 0.59, *χ*^2^ = 21.6, *p* < 0.01), larger irregular rings in MA (50 % SS and 97.5 % SP; *φ* = 0.58, *χ*^2^ = 20.09, *p* < 0.01) and absence of rings in OA-group (99.5 % SS and 87.5 % SP; *φ* = 0.83, *χ*^2^ = 41.57, *p* < 0.001).

#### Inverted colour setting (Table 2; Figs. 2, 3, 4)

##### Morphology of fibers

*Papillary dermis* In the upper part of PD (Fig. 2) the presence of curled thin short fibers was a highly sensitive (95 %) and specific (80 %) feature of YA-skin (*φ* = 0.71, *χ*^2^ = 30.30, *p* < 0.001). Thick curled fibers were highly sensitive (99.5 %) but moderately specific (67.5 %) for OA-skin (*φ* = 0.63, *χ*^2^ = 24.17, *p* < 0.01). Both types of fibers were present in MA-skin in 8/20 (40 %) and 12/20 (60 %) cases, respectively. In the lower part of PD (Fig. 3) the presence of thick, intermediate wavy fibers was highly sensitive (95 %) and specific (85 %) of YA-skin (*φ* = 0.76, *χ*^2^ = 35.11, *p* < 0.001). Thick straight fibers were highly sensitive (99.5 %) but moderately specific (62.5 %) for OA-skin (*φ* = 0.59, *χ*^2^ = 21.06, *p* < 0.01). Both types of fibers were present in MA-skin in 6/20 (30 %) and 14/20 (70 %) cases, respectively. *Superficial reticular dermis.* Coarse intermediate curved rope-like bundles of fibers are 90 % sensitive and 82.5 % specific for YA-skin (*φ* = 0.69, *χ*^2^ = 28.83, *p* < 0.001). Coarse long straight rope-like bundles of fibers were highly sensitive (99.5 %) but poorly specific (62.5 %) for OA-skin (*φ* = 0.59, *χ*^2^ = 20.68, *p* < 0.01). Both bundles of fibers could be observed in MA, in 7/20 (35 %) and 13/20 (75 %) cases, respectively (Fig. 4).

##### Organisation of fibers

*Upper papillary dermis* (Fig. 2). The presence of short loosely interwoven fibers was a highly sensitive (95 %) and specific (85 %) feature of YA-skin (*φ* = 0.76, *χ*^2^ = 35.11, *p* < 0.001). Fibers aggregating in a lace-like network were highly sensitive (99.5 %) but poorly specific (62.5 %) for OA-skin (*φ* = 0.59, *χ*^2^ = 21.06, *p* < 0.01). Both organizations were also observed in MA-skin, in 6/20 (30 %) and 14/20 (70 %) cases, respectively. *Lower papillary dermis* (Fig. 3)—*upper reticular dermis* (Fig. 4). Fibers organized randomly in “feltwork” was a dominant feature of YA-skin (95 % SS, 87.5 % SP; *φ* = 0.79, *χ*^2^ = 37.81, *p* < 0.001). Intermediate long fibers aligned in few directions were predominantly observed in MA-skin although with low sensitivity (65 %) and moderate specificity (82.5 %) (*φ* = 0.48, *χ*^2^ = 13.54, *p* < 0.01). Moreover, this fibre organization was noticed in 1/20 (5 %) YA-skin and 6/20 (30 %) cases of OA-skin. Long fibers aligned in one direction corresponding to furrow pattern were predominantly observed in OA-skin with moderate sensitivity (70 %) and specificity (76 %) (*φ* = 0.62, *χ*^2^ = 23.05, *p* < 0.01). This kind of fibre organization was absent in YA-skin and present in 2/20 (10 %) MA-skin.

##### Clusters of dots in PD

Dispersed dots aligned with fibers or lining hyporeflective holes in a vertical orientation (candle stick holder orientation) were found with high sensitivity (95 %) and specificity (90 %) in YA-skin (*φ* = 0.82, *χ*^2^ = 40.75, *p* < 0.001). In MA-skin an increased density of dots aligned in a more horizontal orientation was observed with moderate sensitivity (80 %) but high specificity (92.3 %) (*φ* = 0.73, *χ*^2^ = 32.2, *p* < 0.001). Compact blotches were exclusively observed in OA-skin (90 % SS, 99.8 % SP; *φ* = 0.92, *χ*^2^ = 50.98, *p* < 0.001).

##### Dermal microvasculature

Features of the dermal microvasculature were assessed both at the PD and RD (Table 3; Figs. 3, 4).

1. *Capillary density in PD* A high capillary density was predominantly observed in YA-skin (95 % SS, 95 % SP; *φ* = 0.89, *χ*^2^ = 47.47, *p* < 0.001). Low capillary density was a highly sensitive and specific feature in OA-skin (90 % SS, 97.5 % SP; *φ* = 0.89, *χ*^2^ = 47.18, *p* < 0.001). In MA-skin an intermediate capillary density was found (85 % SS, 92.5 % SP; *φ* = 0.78, *χ*^2^ = 36.04, *p* < 0.001).
2. *Vessel morphology in PD* The presence of large ovoid hyporeflective spaces was a sensitive (95 %) and specific (87.5 %) feature for YA-skin (*φ* = 0.79, *χ*^2^ = 37.81, *p* < 0.001). Small ovoid hyporeflective spaces were observed in 16/20 (80 %) cases of MA-skin (*φ* = 0.77, *χ*^2^ = 35.71, *p* < 0.001), and only 1/20 (5 %) case of YA-skin and 1/20 (5 %) case of OA-skin. The presence of small pinhole hyporeflective spaces was a sensitive (95 %) and specific (97.5 %) feature of OA-skin (*φ* = 0.93, *χ*^2^ = 51.34, *p* < 0.001).
3. *Vessel morphology in RD* Elongated vessels in horizontal plane were present in 19/20 cases of YA-skin (95 % SS, 95 % SP; *φ* = 0.89, *χ*^2^ = 47.18, *p* < 0.001). Larger elongated vessels in horizontal plane were present in 18/20 cases of MA-skin (90 % SS, 92.5 % SP; *φ* = 0.89, *χ*^2^ = 47.18, *p* < 0.001). Prominent and branched vessels with hyper-reflective cuff were exclusively observed in OA-skins (18/20 cases: 90 % SS, 99.8 % SP; *φ* = 0.89, *χ*^2^ = 47.18, *p* < 0.001).

**Table 3:** Quantitative evaluation of age-related changes in compaction and backscattered intensity (brightness)

Feature	Young aged group	Middle aged group	Old aged group
Compaction of epidermis (µm) Δ_EP_	64.5 [±3.03] (*p* < *0.001*)*	43.5 [±2.02] (*p* < *0.001*)	33.75 [±1.43] (*p* < *0.001*)
Compaction of DEJ (Degrees °) Δ_DEJ_	63.8 [±1.96]	60.2 [±2.22]	35.75 [±1.36] (*p* < *0.001*)
Compaction of papillary dermis (µm) Δ_PD_	63.75 [±3.75] (*p* < *0.001*)	54.75 [±2.51] (*p* < *0.001*)	30.75 [±1.19] (*p* < *0.001*)
Visibility of fibers in deeper reticular dermis (µm) *V*_RD_	79.4 [±3.09]	65.15 [±6.94]	175.2 [±12.16] (*p* < *0.001*)
Brightness of epidermis (AU) *I*_EP_	140.15 [±8.13]	154.05 [±17.12]	447.45 [±48.08] (*p* < *0.001*)
Brightness of DEJ (AU) *I*_DEJ_	47.5 [±8.09] (*p* < *0.001*)	77.95 [±10.88] (*p* < *0.001*)	184.25 [±13.74] (*p* < *0.001*)
Brightness of papillary dermis (AU) *I*_PD_	197.2 [±14.74] (*p* < *0.001*)	270.05 [±20.67] (*p* < *0.001*)	446.1 [±28.23] (*p* < *0.001*)

* *p* values are mentioned whenever appropriate; for details see “Results”

### Quantitative evaluation of IAR changes in compaction and brightness (backscattered intensity) of different skin layers (Figs. 5, 6; Table 3)

1. *Epidermis* A significant difference (*p* < 0.001) in compaction of the epidermis could be observed between the three groups. This compaction increased progressively with age. Regarding the brightness of epidermis a significant (*p* < 0.001) increase could be observed in OA group compared to the other two groups.
2. *Dermo-epidermal junction* A significant increase (*p* < 0.001) in compaction and brightness of the DEJ was observed with age.
3. *Papillary dermis* An age-related significant (*p* < 0.001) increase in compaction and brightness of the papillary dermis was noticed.
4. *Reticular dermis* The depth of visibility of fibers in deeper layers of reticular dermis increased significantly (*p* < 0.001) with age.

## Discussion

In this study we presented morphological features of sun-protected skin visualized in 3-D by HD-OCT in women belonging to three different age groups.

The terminology and study design of the in vivo confocal microscopy studies [30, 51] dealing with skin ageing related morphological changes of epidermis and superficial dermis have been adapted to the HD-OCT. A new terminology and adjusted study design have been proposed regarding morphological assessment of fibers in both papillary and superficial reticular dermis as well as dermal microvasculature in inverted colour setting. Moreover, the present study offers for the first time a quantitative evaluation of HD-OCT descriptors for intrinsic skin ageing based on backscattered intensity measurements.

HD-OCT enables imaging of IAR qualitative skin changes. HD-OCT permits the visualization of the surface texture and furrow pattern in one single en face image because of the large field of view (1.8 × 1.5 mm). This paper suggests that loss of intersecting furrows in sun-protected skin sites is a chronological process, not necessarily linked to sun damage. These findings are in line with previous studies regarding RCM and skin surface topography [1, 30, 51].

With intrinsic ageing, the flattening of the DEJ on cross-sectional HD-OCT imaging is more pronounced. The higher axial resolution (3 µm) of HD-OCT probably enables a better visualization of the DEJ compared to other non-invasive technologies with cross-sectional imaging such as HF-US and conventional OCT. According to Lavker et al. the major change in ageing skin is the flattening of the DEJ because of retraction of the epidermal down-growths in combination with a loss in proliferative capacity associated with the aged epidermis [26]. Age-related functional and structural changes in human DEJ components have been described [27]. The flattening of the DEJ on HD-OCT cross-sectional images corresponds with irregular papillary rings up to the complete disappearance of these rings on en face images. These findings are in line with previous RCM observations by Longo and Wurm et al. [30, 51].

In contrast to MTP [23, 24, 38], collagen fibers cannot be distinguished from elastic fibers by HD-OCT [9]. However, age-related morphological changes of the dermal matrix fibers could be observed with HD-OCT. Moreover, real time 3-D HD-OCT provided volumetric information about the dermal matrix fibers organisation. With intrinsic skin ageing, fibers in PD become thicker, longer, straighter and progressively aggregated in a lace-like network. In the superficial RD these fibers formed IAR straight thick rope-like bundles, no longer oriented randomly but in only in few directions. These findings are in line with evidence for the IAR degradation of fibrous extracellular matrix components including elastin, fibrillin-containing oxytalan fibers and the collagen types I, III and IV [32]. Interestingly only in OA-skin very long (>400 µm), almost unidirectional fibers were imaged by HD-OCT at up to a 185 µm depth under the PD/RD junction. Moreover, these fibers were aligned with the linear furrow pattern. This phenomenon probably corresponds to the process of glycation producing crosslinks between macromolecules, which provides an explanation for the increased age-related stiffness of the skin [35]. In PD, dispersed dark dots (imaged in the inverted colour setting mode) aligned with fibers and vessels and become more and more condensed with intrinsic ageing. In young skin, these dark dots had a candlestick-holder-like 3-D configuration as if they were sustaining the dermal papillae. In elderly skin these black dots condensed progressively to form large dark blotches near the flattened DEJ. To the best of our knowledge, these observations have not been described in studies using other in vivo microscopy techniques. Our findings seem to be in agreement with observations made by scanning electron micrography of matrix fibers in young and aged dermis (low resolution mode) [26]. The present study, however, suggests that flattening of the DEJ is related to the disappearance of the support of dermal papillae by the candlestick-holder-like configuration.

IAR morphological changes of the cutaneous microcirculation were observed by HD-OCT. An age-related decrease in number and size of capillary loops in the dermal papillae and an increase in size of the sub-papillary plexus are in line with laser Doppler flowmetry and videocapillaroscopy findings [22, 28, 29, 47]. Interestingly our study described a hyper-reflective cuff around the branched vessels of the sub-papillary plexus in elderly skin.

A quantitative assessment of HD-OCT descriptors for intrinsic skin ageing has been made possible by ImageJ^®^ software analysis of HD-OCT images. A significant progressive compaction of epidermis, DEJ and PD with age could be detected. This compaction paralleled the increase in backscattered intensity or brightness of the different layers.

An IAR significant progressive compaction of the epidermal component could be found. In the present study, a new method for epidermal thickness (ET) assessment has been described. In this method stratum corneum thickness was included in the ET measurement. ET measurement by HD-OCT has already been discussed in detail by a recent investigation [8]. In that study, the thickness of the stratum corneum was not included in the overall ET assessment of the skin of the back; moreover, 55 % of the subjects belonged to YA-group and 45 % to MA-group. A mean value for ET at the back was 47.38 µm (±1.07 µm 95 % CI). In the present study, the mean value for ET at the inner site of the upper arm of subjects aged between 20 and 60 years was 54 µm (±3.75 µm 95 % CI). The difference between the means of ET in the two studies could be explained by stratum corneum in-/exclusion and anatomic site. The IAR compaction of the epidermis is in agreement with findings by other non-invasive technologies such as RCM [30, 51], MPT [23] and conventional OCT [14]. Interestingly, in the present study a significant increase in epidermal backscattered intensity (brightness) in elderly skin was observed compared to MA- and YA-skin. Skin dryness represents an important characteristic of aged skin. Aquaporin-3 distribution in human epidermis is consistent with epidermal water distribution and parallels the steep water gradient at the junction between stratum granulosum and stratum corneum [3]. A significant decrease of aquaporin 3 (AQP-3) expression in the epidermis with chronological ageing has been described [45] probably explaining the significant increase in epidermal brightness of HD-OCT images with age.

A significant increase in compaction and brightness of the DEJ with chronological age was noticed with HD-OCT. This is in agreement with the IAR degradation of fibrous extracellular matrix components and with the loss of the oligosaccharide fraction which in turn impacts on the ability of tissue to retain bound water [27, 32]. A decreased DEJ thickness with age has already been described for conventional OCT; moreover, there is evidence that the DEJ thickness is higher in African Americans than in Caucasians [39].

With intrinsic ageing, a more compact pattern of the fibrous dermal component of PD could be quantified and correlated with decrease in the voids or areas between the fibers of PD on HD-OCT. These areas correspond most probably to the presence of the ground substance consisting in particular of hyaluronic acid and chondroitin sulphate. The IAR loss of the oligosaccharide fraction impacts on the capacity of the PD to retain bound water [32]. On HD-OCT this loss of bound water resulted in higher brightness. The IAR compaction of the dermis is in agreement with other non-invasive technologies such as MPT [23], conventional OCT [33] and HF-US [16–20, 41, 43].

The highest peak after the valley (Fig. 6) corresponds with the junction between PD and RD as described for conventional OCT [33]. Moreover, the depth of visibility of fibers in reticular dermis increased dramatically in the OA group. This could be explained by fibre rearrangements and alterations such and glycation of collagen fibers [32, 35].

This pilot study has some limitations: (1) only IAR changes in skin morphology have been studied, with no comparison with sun-exposed areas; (2) only women were assessed; (3) individuals with significant systemic comorbidities were excluded and last but not least (4) no histological validation of IAR qualitative skin changes has been performed.

In conclusion, HD-OCT permits to assess qualitatively and quantitatively in vivo and real time three-dimensional IAR morphological skin changes in high resolution from the skin surface to the superficial reticular dermis. This could offer a new possibility to test the efficacy of different cosmetic products. Moreover, HD-OCT assessment of these changes could provide interesting additional information regarding the biological age of the subject as defined by the Framingham CVD risk score [12]. Furthermore, skin wrinkling at the upper inner arm has been linked to health status [48] and elastin morphology in the PD has been linked to cardiovascular diseases risk [37]. These represent interesting topics for future research.
